# Editorial: Towards HRI of everyday life

**DOI:** 10.3389/frobt.2025.1657518

**Published:** 2025-09-01

**Authors:** Karolina Zawieska, Mohammad Obaid, Wafa Johal

**Affiliations:** 1 School of Culture and Society, Aarhus University, Aarhus, Denmark; 2 Department of Computer Science and Engineering, Chalmers University of Technology and University of Gothenburg, Gothenburg, Sweden; 3 School of Computing and Information Systems, University of Melbourne, Melbourne, VIC, Australia

**Keywords:** social robots, human-robot interaction (HRI), everyday life, robots, in the wild studies

Nowadays, it is often assumed that, at least in principle, robots in general, and social robots in particular, have the potential to increase human wellbeing across a wide range of sectors and application areas. At the same time, efforts to introduce such systems into our everyday environments have had only limited success and it is yet to be seen whether the impact of social robots on our daily lives, professional and private, will actually be beneficial. Therefore, there is a need to systematically address and study the long-term use and presence of social robots in everyday environments, where humans not only interact with robots, but also have them integrated into the totality of their lived experiences in everyday life.

One of the main ways to investigate human engagement with social robots in everyday environments is through HRI studies conducted “in-the-wild”. While such an approach has certainly been instrumental and increasingly used in HRI research, in this Research Topic we have proposed to further expand the underlying theoretical and methodological frameworks to address the notion of “everyday life.” Such a notion is viewed here as an important sociological concept and an umbrella term that allows us to address, not only specific application domains for robots, but also the continuity and totality of human lived experiences that emerge through long-term engagement with social robots in everyday life contexts. This is in a situation where placing social robots in everyday settings has often been an explicit goal for robot development but the very notion of “everyday life” has not been systematically analysed. Therefore, this Research Topic has brought together a number of contributions to further develop the HRI domain. While the main audience of this Research Topic is the HRI community, we have welcomed contributions from any disciplinary, interdisciplinary, theoretical, or methodological perspective.

As a result, we have published eight articles centered on the themes of integration, acceptance and people’s attitudes towards robots in everyday life.

Kühne et al. investigates the use of dialects in social robots and its impact on the perceived trustworthiness and competence of robots. This is part of a broader inquiry into design and contextual factors that affect people’s acceptance of robots as social interaction partners. In particular, the study involved playing a short online video featuring NAO the robot speaking either in the Berlin dialect or standard German and asking one hundred twenty native German speakers to evaluate robots through a survey. While the study resulted in observing a slight trend of higher trust and competence evaluation for the standard German-speaking robot compared to the robot that spoke the Berlin dialect, the difference was not statistically significant and the two robots received largely comparable ratings in terms of both competence and trustworthiness. Also, on the one hand, the study found a positive correlation between certain variables, e.g., between participants’ self-reported Berlin dialect proficiency and trustworthiness, and on the other hand, it also depicted a complex picture of people’s perceptions of robots being affected by a number of factors that go far beyond robot features such as demographic characteristics, or a type of device used to watch the video.

Zafrani et al. focuses on the acceptance and assimilation of socially assistive robots (SARs) in everyday life of the elderly persons. It presents the study that examined the uses, constraints and outcomes of engaging with ‘Gymmy’ the robot in real-life conditions and over a long period of time, and their effect on the older adults Quality Evaluations of SARs. The study involved nineteen community-dwelling adults aged 75–97 who interacted with a personal training robot installed at their homes for a period of 6 weeks. The main findings of the study involved identifying two assimilation patterns among the participants who could be categorized either as “Fans” or “Skeptics” based on the type of experience they reported regarding the robot. These two groups differed in terms of the positive vs. negative experience as well as participants’ personal background, attitudes towards robots before and after using the robot in question, and actual user experience. Thus, the study has shown that assimilation and acceptance of SARs is far from being a homogenous process and requires and requires careful consideration of the older adults’ needs and concerns.

Zawieska and Hannibal poses the basis for this Research Topic as it discusses the existing and possible conceptual frameworks for the study of ‘everyday life’ in the HRI research. The paper first provides an overview of the ways everyday life is typically addressed in the HRI studies, namely, in terms of settings, activity, population and/or methodology. Subsequently, the paper proposes further conceptual developments towards a systematic study of everyday life in HRI as a Research Topic in its own right. In the process, it follows Social Sciences and Humanities (SSH) and sociological perspectives that have a long tradition of studying the everyday. In search of possible synergies between the HRI and SSH approaches, the paper builds upon the notion of ‘lived experiences’, and depicts the everyday as an open-ended process. It also engages with a critique of the contemporary everyday life as it calls for challenging the underlying understanding of the everyday and the real-world as “natural” and by doing do, widening the scope of HRI research to include ethico-political stances oriented towards the pursuit of a “good life.”

Komatsu et al. conducted participatory design workshops with middle-to-older adults in Japan—once during the COVID-19 pandemic and again post-pandemic—to explore how their needs and attitudes toward robot technologies evolved due to changing social circumstances. Drawing on Nowland et al.’s “stimulation vs. disengagement” framework, the study found a marked shift over time: during the pandemic, participants prioritized robot tools that enabled distanced communication and social sharing with family, aligning with the stimulation hypothesis. After pandemic restrictions lifted, their focus shifted inward—toward personal wellbeing, ease of use, and technologies that alleviate digital exclusion, reflecting the disengagement hypothesis. Throughout, the participants consistently emphasized the importance of user-friendly, multifunctional robotic solutions—citing familiarity, simplicity, and seamless integration into their daily routines (e.g., combining guidance with communication tools). The authors highlight that Japan’s super-aged context and rapid digitization during COVID-19 exacerbated older adults’ digital exclusion, particularly disadvantaging those less tech-savvy. They conclude with a strong call for inclusive co-design approaches that address evolving social needs, from enabling connection during isolation to supporting personal wellbeing in the post-pandemic era—ensuring robot technologies remain relevant and accessible across life stages and contexts.

Hägglund et al. explore young adults’ trust considerations when interacting with a socially assistive robot in a high-stakes pharmacy scenario—specifically, medication counseling on emergency contraceptive pills. Utilizing a co-creation methodology, they worked with participants to design a prototype application using the Furhat social robot platform. Through in-lab testing and subsequent interviews (six participants), they conducted an inductive, reflexive thematic analysis. The study produced five distinct “tales of trust”, each represented as a persona embodying varying expectations and willingness to trust a robot in this sensitive domain. The research identifies six key factors that drive initial trust formation: physical position (spatial relation to the robot), perceived autonomy, interaction boundaries, feelings of shame, eye gaze, and conversational alignment. These factors shaped whether participants were open to consulting a robot about intimate health matters. By mapping a continuum from low to high trust expectations, the authors illuminate the nuanced interplay of affective, contextual, and design elements influencing user trust in socially assistive robots. The insights significantly inform the understanding of trust formation in HRI, especially in sensitive healthcare contexts, offering important design considerations for socially assistive robotics in the healthcare sector.

Ostrowski et al. present a year-long co-design study involving 28 older adults, aimed at developing HRI guidelines that reflect seniors’ real-world needs. Participants engaged in a series of interviews, design sessions, and reflections to articulate the types of interactions they would want in daily life. These older co-designers expressed growing confidence over time, increasingly favoring transactional robot capabilities—like reminders and scheduling—while maintaining reservations about surveillance-related functions such as personal data tracking and monitoring. Beyond practical assistance, the study found measured enthusiasm for robots facilitating social connection, monitoring bodily signals, and supporting emotional well-being—all tempered by concerns over autonomy, privacy, and the “naturalness” of robotic interactions. Employing ethnographic decision-tree modeling, the authors demonstrated that simpler, non-intrusive features gained acceptance, whereas more invasive ones triggered sustained skepticism. They conclude by offering clear, user-driven guidelines for HRI design with older adults, and highlight several areas—such as ethical data practices, enhancing agency, and improving emotional realism—that warrant deeper investigation before broader deployment.

Vetter et al. conducted a study that explores integrating robotic technologies in the care sector, where such innovations are seen as solutions to labor shortages and aging populations. Their research applied an integrated approach combining value sensitive design and speculative design to explore the complexities of care environments, focusing on the diverse needs, goals, and socio-material arrangements that shape this space. Drawing on six interviews and six card workshops with Austrian care workers and residents, five key themes emerged: trust-building routines, stakeholder negotiations, reciprocal and affective care, caregiver self-care, and material mediation. To provoke reflection and discussion, six speculative vignettes were created, highlighting tensions that arise when technologies disrupt established care practices. The study offers valuable insights for robotic designers to understand care values and dynamics prior to developing interventions.

Irfan et al. conducted a participatory design study with 28 older adults to explore their expectations of conversational companion robots powered by models, such as large language models (LLMs). The study introduced a functioning robot prototype using LLMs in everyday life scenarios to prompt discussion. Through a thematic analysis process of the data, the findings revealed that older adults prefer robots that actively engage in conversations during isolation and passively accompany them in social settings. Key expectations included memory and personalization, privacy and control over data, useful reminders and information, support for social connections, and the expression of empathy and emotion. Based on the findings outlined, the study offers design recommendations for incorporating models (such as LLMs) into companion robots. The outcomes of the articles provide insights that extend beyond robotic agents and contribute broadly to the development of socially responsive conversational agents for older adults.

We hope that with these contributions we have shed light on the importance of the notion of everyday life with robots, and the need for multifaceted HRI approaches that would consider a whole range of factors that together contribute to human lived experiences with robots. The next step is to further develop more nuanced and HRI-specific methods and approaches that would allow us to study a unique phenomenon of life with robots at is unfolds.

